# Optimal Energy Consumption Analysis of Natural Gas Pipeline

**DOI:** 10.1155/2014/506138

**Published:** 2014-05-13

**Authors:** Enbin Liu, Changjun Li, Yi Yang

**Affiliations:** ^1^Southwest Petroleum University, Chengdu 610500, China; ^2^CNPC Key Laboratory of Oil & Gas Storage and Transportation, Southwest Petroleum University, Chengdu 610500, China; ^3^Beijing Oil and Gas Control Center, Beijing 100191, China

## Abstract

There are many compressor stations along long-distance natural gas pipelines. Natural gas can be transported using different boot programs and import pressures, combined with temperature control parameters. Moreover, different transport methods have correspondingly different energy consumptions. At present, the operating parameters of many pipelines are determined empirically by dispatchers, resulting in high energy consumption. This practice does not abide by energy reduction policies. Therefore, based on a full understanding of the actual needs of pipeline companies, we introduce production unit consumption indicators to establish an objective function for achieving the goal of lowering energy consumption. By using a dynamic programming method for solving the model and preparing calculation software, we can ensure that the solution process is quick and efficient. Using established optimization methods, we analyzed the energy savings for the XQ gas pipeline. By optimizing the boot program, the import station pressure, and the temperature parameters, we achieved the optimal energy consumption. By comparison with the measured energy consumption, the pipeline now has the potential to reduce energy consumption by 11 to 16 percent.

## 1. Introduction

Gas pipelines are the bond that connects gas production and consumption; therefore, their operation must be safe, smooth, and effective. In 1961, a US gas pipeline company collaborated with IBM to simulate and optimize the operation of gas pipelines [1]. This represented the prelude to additional optimal operation research on gas transmission pipelines.

In 1983, Goldberg introduced a genetic algorithm, which was one of the most popular optimization algorithms of the time, to optimize the operation of a natural gas pipeline [2]. The optimal solution of this optimization model considered the minimum energy consumption to be the objective function and promoted research on long-distance pipeline operation optimization using intelligent optimization algorithms. Between 1984 and 1997, many scholars, such as Mantri, Renji, Bhaduri, Anglard, Wilson, Ryan, and Berry et al., continued to improve the operation optimization model of gas transmission pipelines, as well as the methods for obtaining solutions [3–10]. In 1998, Carter took advantage of the dynamic programming algorithm for constructing a steady-state operation optimization model of a gas transmission pipeline [11]. Based on his calculations, he concluded that the dynamic programming algorithm converged faster than the annealing and genetic algorithms. By the end of the 20th century, network simulation models and the optimization of the operation technology for natural gas transmission pipelines had reached maturity. The nonlinear operation optimization model for long-distance gas transmission pipelines (including a discrete variable and objective function for minimum energy consumption) had also been recognized. Since then, researchers have made a sustained effort, taking into consideration the various aspects of the optimization algorithm, to solve the network operation optimization model for a gas transmission pipeline more quickly and effectively. For example, in 2000, Sun and others established a comprehensive pipeline operation optimization expert system [12]. This expert system was capable of detecting the pipeline filling state such that the system could decide the control requirements. It was also able to work out the demand of the corresponding energy consumption. Based on these two steps, a fuzzy model can be used to determine the exact extent to which the compressor should be open. In 2002, Cobos-Zaleta and Rios-Mercado used the equation relaxation and expansion valve method to solve the operation optimization model for a gas pipeline [13]. In 2004, Rusnak et al. used the steady optimization simulator for dynamic optimization analysis of long-distance pipelines, with the goal of simulating the minimal energy consumption [14, 15]. After 2008, Yi et al. studied the problem of steady-state optimization operation of a main gas transmission pipeline network under a determined throughput. In these studies, the optimal rule was adopted based on the minimum energy consumption cost [16–19].

In this paper, we aim to characterize long-distance natural gas pipeline operation management. For a given throughput, with the minimum pipeline operation energy consumption as the goal, the gas pipeline optimal operation model can be established. This model is solved using a dynamic programming method to obtain the best operation scheme and the minimum energy consumption for the natural gas pipeline.

## 2. Minimum Energy Consumption Prediction Model of a Natural Gas Pipeline

Natural gas pipeline systems are complicated. They are composed of pipelines, stations, compressors, fluids, external environmental factors, and other components. Based on the Chinese policy for energy savings and emission reduction and the premise of the transportation quantity plan (intake quantity or delivery quantity), the pipeline operation department must configure each station's compressors and determine the operating parameters for each station to reach the lowest energy consumption for the pipeline system.

To study the minimum energy consumption of a natural gas pipeline system, we need to establish a corresponding mathematical model. A reasonable and accurate mathematical model is the key to obtaining the best results.

### 2.1. The Objective Function

During operation, the pipeline's main energy consumption is from the compressor's drive. Therefore, we established an objective function as the goal for minimum production unit consumption, which is expressed as

(1) $$\begin{array}{c} \min F=\frac{\left(S_{p}\omega_{1}+S_{g}\omega_{2}\right)}{T_{ur}}, \end{array}$$

where *F* is the production unit consumption of the pipeline in kgce/(10^7^Nm³·km), *S*_*p*_ is the power consumption in kW·h, *S*_*g*_ is the gas consumption in m^3^, *ω*_1_ is the electric coal conversion coefficient based on the Chinese National Standard GB2589-81 of 0.1229 kgce/(kW·h), *ω*_2_ is the gas coal conversion coefficient based on the Chinese National Standard GB2589-81 of 1.33 kgce/m^3^, and *T*_*ur*_ is the turnover in 10^7^ Nm³·km.

The power consumption *S*_*p*_ can be expressed as follows:

(2) $$\begin{array}{c} S_{p}=\overset{n}{\underset{i=1}{\sum }}\frac{N_{i}t_{p}}{\eta_{ei}}. \end{array}$$

The gas consumption *S*_*g*_ can be expressed as

(3) $$\begin{array}{c} S_{g}=\overset{n}{\underset{i=1}{\sum }}\frac{N_{i}t_{p}}{\eta_{gi}}ge, \end{array}$$

where *n* is the number of compressors, *N*_*i*_ is the shaft power of the* i*th compressor in kW, *t*_*i*_ is the running time of the* i*th compressor in h, *η*_*ei*_ is the drive motor efficiency of the* i*th compressor, *η*_*gi*_ is the turbine efficiency of the* i*th compressor, and *ge* is the gas loss rate of the gas turbine in Nm^3^/ /(kW·h).

The turnover *T*_*ur*_ can be expressed as

(4) $$\begin{array}{c} T_{ur}=10^{-4}\overset{n}{\underset{i=1}{\sum }}Q_{i}L_{i}t, \end{array}$$

where *Q*_*i*_ is the volume flow of the* i*th section of the pipeline in Nm^3^/d, *L*_*i*_ is the length of the* i*th section of the pipeline in km, and *t* is the delivery time in d.

### 2.2. Optimization Variables

The power of the compressor depends on the compression ratio, flow rate, and temperature. Because the inbound traffic of the compressor station is known, the power of the compressor can be simplified into a function of the pressure ratio and temperature. The compressor inlet and outlet temperatures depend on the compression ratio; therefore, the optimization variables can be converted into the compression ratio and thus can be converted into the outbound pressure. The optimization variables of the optimization model, that is, the outbound pressures and the boot number, can be expressed as

(5) $$\begin{array}{c} X_{k}=\left(P_{dk},O_{i}\right), \end{array}$$

where *P*_*dk*_ is the outbound pressure of the *k*th compressor station and *O*_*i*_ is the boot number of the *i*th compressor station.

### 2.3. Constraint Condition

To guarantee the safe operation of the pipeline and the devices, both the operation parameters of the pipelines and the operation parameters of the devices must be within the permitted range. Namely, the parameters must be satisfied with a series of constraint conditions.

(*1) Inlet and Outlet Pressure Constraint*. According to the user's need, there are some requirements for the pressures of the subair node. These are expressed as

(6) $$\begin{array}{c} P_{i\min}\leq P_{i}\leq P_{i\max}\;\left(i=1,2,\ldots ,n_{S}\right), \end{array}$$

where *P*_*i*_ is the pressure of the *i*th node in Pa, *P*_*i*__min⁡_ is the minimum permissible pressure of the* i*th node in Pa, and *P*_*i*max⁡_ is the maximum allowable pressure of the *i*th node in Pa.

(*2) Pipeline Strength Constraints*. To ensure the safe operation of the pipelines, the gas pressure must be less than the maximum allowable operating pressure such that

(7) $$\begin{array}{c} P_{k}\leq P_{k\max}\;\left(k=1,2,\ldots ,n_{p}\right), \end{array}$$

where *P*_*k*_ is the pressure of the *k*th pipe in Pa and *P*_*k*max⁡_ is the maximum allowable pressure of the *k*th pipe in Pa.

(*3) Compressor Performance Constraints*. The compressor power equation is

(8) $$\begin{array}{c} N=\frac{MH}{\eta }, \end{array}$$

where *M* is the overflow rate of the compressor in kg/s, *H* is the polytropic head of the compressor, and *η* is the efficiency of the compressor.

The head curve is calculated according to

(9) $$\begin{array}{c} -H=h_{1}S^{2}+h_{2}SQ+h_{3}Q^{2}, \end{array}$$

where *h*_1_, *h*_2_, and *h*_3_ are the fitting coefficients of the head curve, *S* is the speed of the compressor, and *Q* is the actual overflow rate of the compressor in m^3^/d.

The efficiency curve is calculated according to

(10) $$\begin{array}{c} \frac{-H}{\eta }=e_{1}S^{2}+e_{2}SQ, \end{array}$$

where *e*_1_ and *e*_2_ are the fitting coefficients of the power curve.

The buzz curve is calculated according to

(11) $$\begin{array}{c} Q_{\text{surge}}=s_{1}+s_{2}H, \end{array}$$

where *Q*_surge_ is the surging flow in m^3^/d and *s*_1_ and *s*_2_ are the fitting coefficients of the buzz curve.

The stagnation curve is calculated according to

(12) $$\begin{array}{c} Q_{\text{stone}}=s_{3}+s_{4}H, \end{array}$$

where *Q*_stone_ is the stagnation flow in m^3^/d and *s*_3_ and *s*_4_ are the fitting coefficients of the stagnation curve.

From (9) to (12) are plotted in the figure, forming a closed area. This area is the operating area of the compressor.

(*4) Compressor Power Constraints*. The power constraints are represented by

(13) $$\begin{array}{c} N_{\min}<N<N_{\max}, \end{array}$$

where *N*_min⁡_ is the minimum allowable power of the compressor in MW and *N*_max⁡_ is the maximum allowable power of the compressor in MW.

(*5) Compressor Speed Constraints*. The speed constraints are represented by

(14) $$\begin{array}{c} S_{\min}<S<S_{\max}, \end{array}$$

where *S*_min⁡_ is the minimum speed of the compressor in rpm/min and *S*_max⁡_ is the maximum speed of the compressor in rpm/min.

(*6) Compressor Outlet Temperature Constraints*. The temperature constraints are represented by

(15) $$\begin{array}{c} T_{H}<{T_{H}}_{\max}, \end{array}$$

where *T*_*H*__max⁡_ is the maximum outlet temperature of the compressor in K.

(*7) Pipeline Pressure Drop Equation*. The pressure of the pipeline is determined by two factors: the value of the frictional pressure drop and the pressure change due to the elevation change. The calculation of the pressure drop is based on the continuity and momentum equations. Introducing the mass flow rate of the gas [20], we obtain

(16) $$\begin{array}{c} M=\frac{\pi }{4}\sqrt{\frac{\left[P_{Q}^{2}-P_{Z}^{2}\left(1+a\Delta h\right)\right]D^{5}}{\lambda \text{ZRTL}\left(1+\left(a/2L\right)\sum_{i=1}^{n}\left(h_{i}-h_{i-1}\right)L_{i}\right)}}, \end{array}$$

where *M* is the flow of the gas through the pipes in kg/s, *P*_*Q*_ is the starting pressure of the pipeline in Pa  (*P*_*Q*_ = *P*_*d*_), *P*_*Z*_ is the end pressure of the pipeline in Pa  (*P*_*Z*_ = *P*_*s*_),  *T* is the average of the gas flow temperature in K, *L* is the length of the pipeline in m, *D* is the diameter in m, Δ*h* is the elevation difference between the start and end of the pipeline in m, *Z* is the gas compressibility (i.e., the pressure computation of the BWRS state equation), and *λ* is the friction factor.

(*8) Pipe Temperature Drop Formula*. The pipe temperature drop is calculated according to

(17) $$\begin{array}{c} T=T_{0}+\left(T_{Q}-T_{0}\right)e^{-ax}, \end{array}$$

where *T* is the temperature of length *x* of the pipeline in K, *T*_0_ is the temperature of the pipeline where it is deeply buried in K, and *T*_*Q*_ is the temperature at the start of the pipeline in K.

(*9) Pipe Network Node Flow Balance Constraints*. For a natural gas pipeline, in any node, according to the law of conservation of mass, the inflow and outflow of the gas should be 0. In general, for a natural gas pipeline network system with* Nn* node, the gas flow equilibrium equations of the node can be written as

(18) $$\begin{array}{c} \overset{Nn}{\underset{\begin{array}{c} k\in C_{i}\\ i=1 \end{array}}{\sum }}\alpha_{ik}M_{ik}+Q_{i}=0, \end{array}$$

where *C*_*i*_ is the set connected to the* i*th node element, *M*_*ik*_ is the absolute value of element *k* into/out of the node flow connected to the* i*th node, *Q*_*i*_ is the flow in the node exchange with the outside world (flow into the positive, flow out of the negative), and *a*_*ik*_ is the coefficient (when traffic flows in, the *k* node components are +1 and when traffic flows out, the *k* node components are −1).

The mathematical model can be written in the standard form for optimization models as

(19) min⁡ f(x) s.t:   gi(x)≤0 (i=1,2,…,m),

where *x* represents the optimization variables and *m* is the number of constraints.

## 3. Method for Modeling Based on Dynamic Programming

The gas pipeline branch is simplified to a point. The operation process of the pipeline can be regarded as a multistage process. Thus, we can use a dynamic programming algorithm to distribute the optimal ratio of the compressor stations (i.e., the optimal discharge pressure).

Suppose the number of compressor stations is *n* when establishing the dynamic programming model. Treat the gas transmission process from the compressor station of the (*k* − 1)th to the *k*th as the*k*th phase of the correspondence problem. The *k*th stage of the state variables *X*_*k*_ (corresponding to the starting point of the state) is the discharge pressure *P*_*d*,*k*−1_ of the *k*th station. The phase effect for the* k*th station energy consumption (i.e., the power, as shown in formula (1)), with respect to the pipeline total energy consumption of the optimization goal, can build the optimized dynamic programming model of the pipeline's compressor station pressure ratio.

The algorithm for solving the model is composed of the following components: “determine the state space,” “recursive between stations,” “recursive within the station,” and “backtracking algorithm.”

### 3.1. Determine the State Space

In the dynamic programming algorithm, a certain compressor station out of all of the feasible discharge pressures is the state space. The upper boundary of the state space can give the design pressure of the pipeline. The lower boundary, also called the lowest discharge pressure, is difficult to determine. If it is too large, it will increase the unnecessary computation; however, if it is too small, it may miss the optimal solution. We calculated the lowest discharge pressure for the previous compressor station with the limitations of the lowest discharge pressure of this compressor station.

The compressor with the gas turbine or motor drive performs stepless speed regulation, so that the discharge pressure of the compressor station can be within the scope of feasible continuous change. Thus, we must process the state space to obtain the finite state point. In this paper, the outlet pressure range of each compressor station is divided into 300 points to determine the compression ratio of the space.

When the pipeline is running with low throughput, the station operation plan is always run more economically than with a low compression ratio. This must be taken into consideration for circumstances where the pressure is above the permitted level for one of the compressor stations. By setting each station's entrance pressure as part of the state space, the state transition will not leak.

### 3.2. Recursive between Stations

Recursion between stations is a calculation through which the entrance condition of the next compressor station is determined by the outlet condition of the current compressor station, which mainly involves hydraulic and thermodynamic calculation between stations. On the basis of a certain outlet pressure of the compressor station, (16) and (17) can be used to calculate the pressure and temperature at the ends of the pipeline. This provides the inlet pressure and the temperature of the next station.

**Figure 1:**
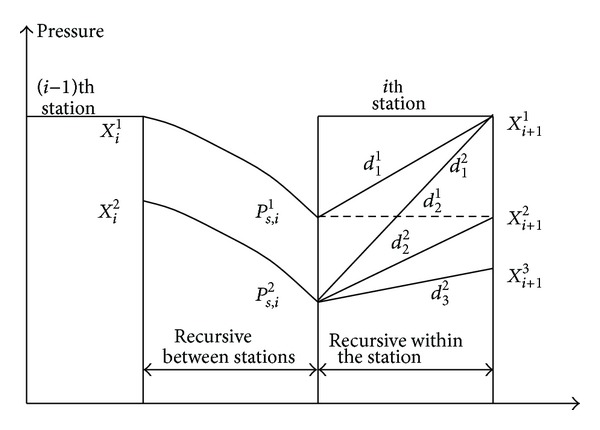
The recursive process.

Taking the recursive between stations shown in Figure 1 as an example, use number (*i* − 1) station's operation condition corresponding to output pressure *X*_*i*_^1^ to recursive between stations to obtain the* i*th station outlet condition corresponding to the inlet pressure *P*_*s*,*i*_^1^. The main steps are as follows. Carry out the pipeline's hydraulic and thermodynamic calculation between the* i*−1th station and* i*th station. The starting point's parameters are *Q*_*d*,*i*−1_^1^, *P*_*d*,*i*−1_^1^, *T*_*d*,*i*−1_^1^. The flow should provide the corresponding changes if there is an injection or disengagement point. The final figures for flow, pressure, and temperature are obtained from the inlet operation. In the end, the optimal index *C*_*d*,*i*−1_^1^ corresponding to *X*_*i*_^1^ should be recorded as the energy consumption of the inlet operation, which reflects the pipeline's energy consumption under the optimal operation scheme from the beginning to the *i*th station.

### 3.3. Recursive within the Station

The recursive within the station gives the outlet station's operation based on the compressor station's inlet operation, which is dominated by the state transfer. For the state before the transfer, in addition to determining the state space, the feasible compression ratio range of compression for every inlet condition should also be obtained, based on the constraint conditions of the decision variables.

Taking the recursive within the station shown in Figure 1 as an example, for *X*_*i*+1_^1^, the method of state transition is as follows. Inspect whether the path from *P*_*s*,*i*_^1^ to *X*_*i*+1_^1^ is feasible, which indicates whether *d*_1_^1^ gained by *X*_*i*+1_^1^ divides *P*_*s*,*i*_^1^ (station pressure ratio, namely, the decision variables) is in line with the pressure ratio range inlet condition *X*_*i*+1_^1^. If not, make the energy consumption of the path a maximum value; otherwise, call for station optimization to obtain the compressor station's optimal scheme under the condition of *X*_*i*+1_^1^ corresponding to the inlet condition and the station pressure ratio of *d*_1_^1^ and obtain the energy consumption of the station at the program (if *d*_1_^1^ is equal to zero, then so is the energy consumption), namely, the stage effect of the stage. The stage effect and *P*_*s*,*i*_^1^ of the corresponding inlet condition recorded from the beginning to the energy consumption of this station are added. Then, the total energy consumption from *P*_*s*,*i*_^1^ to *X*_*i*+1_^1^ can be obtained.

Use the same method to calculate the total energy consumption from *P*_*s*,*i*_^2^ to *X*_*i*+1_^1^. Compared with the former, the smaller one is the *X*_*i*+1_^1^ state transfer result.

### 3.4. Backtracking Algorithm

After the completion of the recursive within the station, we will obtain all of the total energy costs corresponding to several inlet conditions in the terminal station. To obtain the operation program within the minimum energy consumption limit to meet the terminal station's pressure, backtracking of the whole scheme is required.

Backtracking is performed according to the compression station's inlet and outlet operations recorded in the optimal program to determine the optimal operation scheme of the pipeline. Backtracking starts from the gate station's optimal inlet condition, according to every state transfer's recorded results, to find out every compressor station's outlet condition corresponding to the last station's outlet condition.

## 4. Operation Optimization of the XQ Gas Pipeline

### 4.1. Basic Parameters of the XQ Gas Pipeline

#### 4.1.1. Pipe Parameters

**Figure 2:**
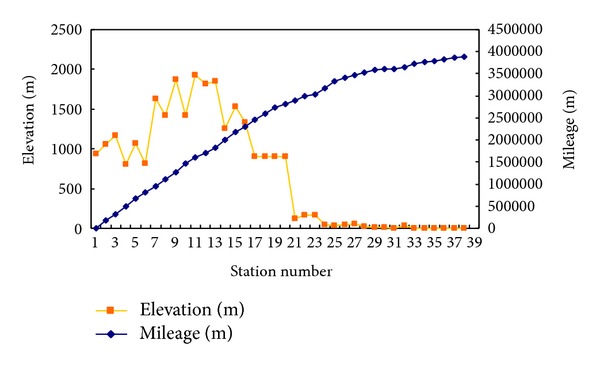
Elevation and mileage of the XQ gas pipeline.

The length of the pipeline is 3840 km, the design capacity is 170 × 10^8^ Nm^3^/year, the design pressure is 10 MPa, and the diameter is Φ1016 × 17.5 mm. The elevation and mileage of the XQ gas pipeline are shown in Figure 2. We can see that the elevation change is large, with the highest point at 1900 m and the lowest point at 1 m.

There are 40 stations in the XQ gas pipeline, including 22 compressor stations and 18 distribution stations, as listed in Table 1.

#### 4.1.2. The Compressor Performance Curve

There are two manufacturers for the compressors used in the XQ gas pipeline (GE and RR). Part of the compressor's coefficients for (9)–(12) is shown in Table 2.

#### 4.1.3. Constraint Conditions

The maximum outbound pressure is 9.8 MPa, while minimum pitted pressure is 5 MPa. The minimum pitted temperature is 15°C, while the maximum outbound temperature is 65°C.

### 4.2. Optimization Research and Analysis

Take the parameters in May 2012 as an example for the optimization calculation. The pitted pressure of the first station is 6.5 MPa and the temperature is 15°C. Each station's gas transmission capacity is shown in Table 3. There are 5 points for admission and 37 points distributed along the line. Through 50 iterations, the optimum operation is determined, as shown in Table 4, for 23 running compressors. Compressors are connected in parallel at all stations. By means of the energy consumption amount, the energy consumption of the scheme is shown in Table 5. The unit consumption for production is 138.37 kgce/(10^7^ Nm³·km), and the actual measurement of energy consumption is lower by −12.70% compared with the same month, indicating that the pipeline has great potential for saving energy.

Using the same method to optimize the operation for 1–7 months in 2012, the energy consumption optimization results can be obtained. As shown in Table 6, 1–3 months is the gas use peak in the winter. The first station's intake is approximately 4800 × 10^4^ Nm³ per day at full load. The period from 4 to 7 months without heating gas is the low point. The first station's intake is approximately 3500 × 10^4^ Nm³ per day, according to the optimal operation scheme proposed in this paper.

We can acquire the operating parameters through the SCADA systems of the pipeline, including the gas consumption and electricity consumption. Therefore, we can obtain the actual energy of the pipeline in Table 6.

**Figure 3:**
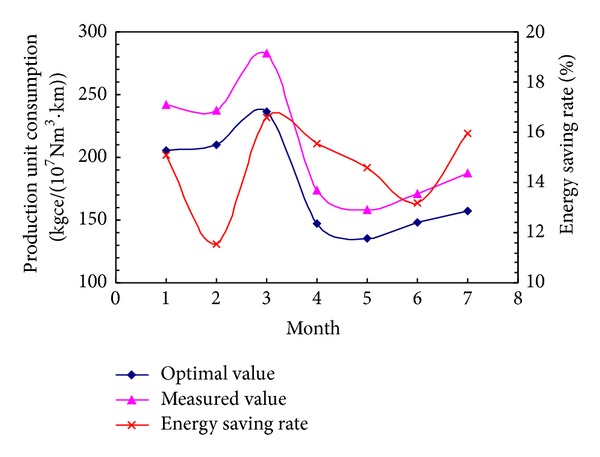
Energy analysis.

The data in Table 6 is plotted in Figure 3. Compared with the measured values, the production unit consumption can be reduced by approximately 11%~17%. Therefore, the pipeline has great energy-saving potential.

## 5. Conclusions

Our conclusions are as follows.

1. Based on a full understanding of actual demands of a pipeline company, we introduce production consumption indicators to establish an objective function of the minimum energy consumption of the gas pipeline and use dynamic programming to solve the model quickly and efficiently.
2. When setting the constraints, it is necessary to consider the pipeline, station, power equipment, topography, and climate and to simplify these constraints reasonably such that the mathematical model can accurately describe not only the energy consumption of crude oil pipeline but also the convenient mathematical operations.
3. According to the dynamic programming method, we compiled the natural gas pipeline running optimization software, which can be used to guide the natural gas pipeline running program analysis and optimize the energy savings. Through the optimization analysis of the XQ nature gas pipeline with the actual working condition, we discovered that the optimal operation scheme can reduce energy consumption by 11%~16%.

## Figures and Tables

**Table 1:** Equipment at each station.

Station		Compressor		Drive type
Number	Type	Model	Number	
1	Compressor	1	2	Gas
2	Compressor	2	2	Gas
3	Compressor	3	1	Gas
4	Compressor	4	2	Gas
5	Compressor	5	2	Gas
6	Compressor	6	1	Gas
7	Compressor	7	2	Gas
8	Compressor	8	2	Gas
9	Compressor	9	2	Electric
10	Compressor	10	2	Gas
11	Compressor	11	2	Gas
12	Compressor	12	1	Gas
13	Compressor	13	1	Gas
14	Distribution			
15	Compressor	14	1	Gas
16	Compressor	15	2	Gas
17	Compressor	16	1	Gas
18	Distribution			
19	Compressor	17	1	Gas
20	Compressor	18	2	Electric
21	Distribution			
22	Compressor	19	2	Electric
23	Distribution			
24	Compressor	20	2	Electric
25	Distribution			
26	Compressor	21	2	Electric
27	Distribution			
28	Distribution			
29	Compressor	22	2	Gas
30	Distribution			
31	Distribution			
32	Distribution			
33	Distribution			
34	Distribution			
35	Distribution			
36	Distribution			
37	Distribution			
38	Distribution			
39	Distribution			
40	Distribution			

**Table 2:** Coefficients for the equation for the compressor performance curves.

Model	*H*_1_	*H*_2_	*H*_3_	*e*_1_	*e*_2_	*S*_1_	*S*_2_	*S*_3_	*S*_4_
1	−0.000282	−0.000393	0.000090	−0.001170	0.000144	4620	0.396	8310	1.44
2	−0.001200	0.000167	0.000045	−0.001470	0.000332	3840	0.145	4910	0.533
3	−0.000403	−0.000348	0.000064	−0.001440	0.000140	5920	0.412	10700	1.47
4	−0.001200	0.000167	0.000045	−0.001470	0.000332	3840	0.145	4910	0.533
5	−0.000390	−0.001090	0.000334	−0.002180	0.000392	3080	0.145	5970	0.585
6	−0.000640	0.000012	0.000023	−0.000883	0.000141	5010	0.342	8520	1.26
7	−0.000183	−0.001100	0.000314	−0.001990	0.000362	3260	0.173	6270	0.79
8	−0.001190	0.000161	0.000042	−0.001450	0.000317	3610	0.149	4640	0.554
9	−0.000644	−0.000679	0.000252	−0.001790	0.000324	2970	0.165	5340	0.504
10	−0.001190	0.000161	0.000042	−0.001450	0.000317	3610	0.149	4640	0.554

**Table 3:** Transmission capacity, 10^4^ Nm³/d.

Station number	Injection volume	Distribution volume
1	3552	0
14	0	291
15	1277	0
21	0	35
22	188	0
23	0	158
24	0	379
25	219	220
26	0	589
27	0	55
28	0	68
29	0	130
30	0	39
31	0	351
32	817	56
33	0	522
34	0	66
35	0	416
36	0	149
37	0	183
38	0	751
39	0	508

**Table 4:** Optimal operation scheme.

Station number	Pitted pressure, MPa	Outbound pressure, MPa	Pitted temperature, °C	Outbound temperature, °C	Compressor boot program
1	6.5	8.43	15	37.28	1 set
2	6.41	9.08	6.61	35.87	2 set
3	7.78	9.75	8.69	27.56	1 set
4	8.5	8.5	6.32	6.32	0 set
5	6.62	9.17	5.09	32.36	2 set
6	8.17	9.78	8.24	23.14	1 set
7	7.96	9.8	6.88	24.09	1 set
8	8.73	8.73	6.73	6.73	0 set
9	6.98	9.8	5.15	33.56	2 set
10	8.54	8.54	6.35	6.35	0 set
11	6.85	9.24	5.15	30.07	2 set
12	8.43	9.8	9.73	22.27	1 set
13	8.81	8.81	7.54	7.54	0 set
15	7.84	9.76	5.18	23.15	1 set
16	7.03	9.7	6.93	33.98	2 set
17	8.05	9.8	11.8	28.32	1 set
19	8.05	9.8	9.41	25.8	1 set
20	7.95	9.74	9.44	26.35	1 set
22	7.68	9.34	8.9	25.18	1 set
24	7.5	9.26	8.36	25.87	1 set
26	7.24	9.03	7.37	25.72	1 set
29	6.29	7.84	5.24	23.39	1 set

**Table 5:** Energy consumption of the optimal operation scheme.

Turnover	452892.63 × 10^7^ Nm³·km
Gas consumption	4154.5 × 10^4^ Nm³
Gas unit consumption	91.7 Nm³/(10^7^Nm³·km)
Production unit consumption	135.4 kgce/(10^7^Nm³·km)
Power consumption	4195 × 10^4^ kW·h
Total energy consumption	61314.19 tce
Power unit consumption	108.9 kW·h/(10^7^ Nm³·km)

**Table 6:** XQ1 energy consumption.

Month	Production unit consumption, kgce/(10^7^ Nm³·km)			Turnover, 10^7^ Nm³·km	Power consumption, 10^4^ kW*·*h			Gas consumption, 10^4^ Nm³		
	Optimal value	Measured value	Energy saving rate		Optimal value	Measured value	Deviation	Optimal value	Measured value	Deviation
1	205.1	241.46	−15.07%	521623	5446	5302	2.72%	7540	8980	−16.04%
2	210.1	237.6	−11.56%	496704	4944	5825	−15.12%	7391	8335	−11.33%
3	236.0	283	−16.59%	476826.49	5267	6996	−24.71%	7976	9529	−16.30%
4	147.0	174	−15.53%	452892.63	4449.69	5345	−16.75%	4594	4377	4.96%
5	135.4	158.5	−14.58%	452892.63	4930.3	4195	17.53%	4154.5	5011	−17.09%
6	148.6	171.2	−13.20%	463643.35	5024.59	5194	−3.26%	4716.23	5487	−14.05%
7	157.4	187.3	−15.97%	486394.54	5049.2	4612	9.48%	5289.32	6424	−17.66%
